# Supplementary material for: Hardware-aware training for large-scale and diverse deep learning inference workloads using in-memory computing-based accelerators
Source: Nat Commun. 2023 Aug 30;14:5282. doi: 10.1038/s41467-023-40770-4 (PMC10469175; doi:10.1038/s41467-023-40770-4)
Supplement: Supplementary file 1 — Supplementary Information [file 41467_2023_40770_MOESM1_ESM.pdf]

Hardware-aware training for large-scale and diverse deep learning inference workloads using in-memory computing-based accelerators

Malte J. Rasch*[1], Charles Mackin[2], Manuel Le Gallo[3], An Chen[2], Andrea Fasoli[2], Frédéric Odermatt[3], Ning Li[1], S.R. Nandakumar[3], Pritish Narayanan[2], Hsinyu Tsai[2], Geoffrey W. Burr[2], Abu Sebastian[3], and Vijay Narayanan[1]

[1]*IBM Research, TJ Watson Research Center, Yorktown Heights, NY USA*
[2]*IBM Research Almaden, 650 Harry Road, San Jose, CA USA*
[3]*IBM Research Europe, 8803 Rüschlikon, Switzerland*

August 2, 2023

Contents

A Supplementary Methods **2**
A.1 Details on the HWA training simulations 2

B Supplementary Notes **6**
B.1 Static input range learning versus dynamic scaling 6
B.2 Generalizing to other memory technologies 7
B.3 Tile-wise digital instead of column-wise digital output scale 10
B.4 Column-wise noise impact for ImageNet CNNs 13

List of Figures

1 Generalization of the AIMC model to ReRAM 8
2 Compression of column-wise output scales 11

List of Tables

1 AIMC crossbar model parameters 2
2 HWA training methods comparison 2
3 Hyper-parameters for HWA training 6
4 Inference of using ReRAM-based AIMC crossbar model 9
5 Weight injection noise distributions 10
6 Inference results for FP-trained DNNs using tile-wise output scales 10
7 Training with tile-wise output scales 12
8 PCM noise-exempt columns for ImageNet CNNs 13

A Supplementary Methods

Parameter description	value	AIHWKIT configuration name
ADC precision	8 bit	`forward.out_res`
DAC precision	8 bit	`forward.inp_res`
System noise referred to output	0.04	`forward.out_noise`
Output bound	10.0	`forward.out_bound`
Input bound	learned	`forward.inp_bound`
(Max) tile size	512×512	`mapping.max_input_size`
Wire-conductance to $\hat{g}_{\max}$ ratio	571428.57	`forward.ir_drop_g_ratio`
Short-term weight noise	0.0175	`PCM_READ`, `forward.w_noise`
IR-drop scale	1.0	`forward.ir_drop`
PCM noise and drift	data-calibrated [3]	`PcmLikeNoiseModel`
Drift compensation	global	`GlobalDriftCompensation`
Layer bias	digital	`mapping.digital_bias`
Digital output scale	column-wise	`mapping.out_scale_columnwise`

Supplementary Table 1: **AIMC crossbar model parameters**. Parameter of our Standard PCM analog in-memory computing (AIMC) model for inference (see also Manuscript Fig. 2 and Manuscript Fig. 3). The parameter names of the configuration settings of the open-source package IBM Analog Hardware Acceleration Toolkit (AIHWKit) are indicated [1, 2]. Noise management and bound management techniques [4] are turned off and instead the learned input range is taken.

Hardware-aware (HWA) training method	1 sec	1 hour	1 day	1 year
HWA method from [5] adapted	6.79 0.01	7.27 0.02	7.80 0.02	9.60 0.05
our HWA training (avg 3 training trials)	6.79 0.02	7.08 0.03	7.50 0.04	8.36 0.06
FP_{32} mapped to analog (no HWA training)	10.03 0.18	11.94 0.35	14.27 0.39	22.63 1.07
only output noise	7.04 0.05	7.40 0.07	8.03 0.09	10.33 0.25
only short-term weight noise	7.01 0.04	7.62 0.09	8.15 0.12	10.47 0.38
no noise (but other nonidealities)	7.36 0.05	8.04 0.06	8.67 0.18	11.38 0.25

Supplementary Table 2: **HWA training methods comparison.** Validation of our hardware-aware (HWA) training methods and a number of ablations for a ResNet-32 model on CIFAR10 [5]. Average inference test errors are in percent (mean over 25 inference trials, and standard error of the mean). For comparison, we obtained the same weights as in [5], but mapped them to our modified hardware-model with additional noise sources (see Manuscript Eq. 1 and Manuscript Eq. 2) Our method performs results in a $> 10\%$ more robust model (for the most challenging t_{eval}) validating our approach. Note that we here do not use the learning rate schedule nor the Gaussian weight clipping suggested by [5], nor any best checkpoint selection beyond hyper-parameter tuning (added noise strength and learning rate). Note that it is crucial to add weight noise, however, as was also done in [5].

A.1 Details on the HWA training simulations

A.1.1 LSTM on Penn Treebank Dataset

Here the deep neural network (DNN) model is a two-layer long-short-term memory (LSTM) with a hidden size of 650 for word-based prediction on the Penn Treebank Dataset (PTB) [6] using cross entropy loss. The encoder layer is implemented digitally, whereas the decoder layer is assumed to be on AIMC and consists of 10K classes – each corresponding to a word in the dictionary. We performed HWA training generally as described in 'AIMC hardware aware DNN training' with the following particularities. The HWA model is trained for 60 epochs using a conventionally floating point (FP) trained model as a starting point. We scan several hyper-parameters and find the best HWA-trained

model (based on the average of 3 training trials) to have a base learning rate of 0.01, a learning rate decay of 0.9 (applied after each epoch if the test error is not improving on the validation set; also tested 0.75 which performed slightly worse), dropout ratio of 50%, injection of the expected PCM noise (at $t = 20$s without drift) each mini-batch scaled by 5 (ramping up over the initial 1000 batches), stochastic gradient descent (SGD) momentum of 0.9, and maximum gradient norm of 10. We use a weight decay (L2 regularization) factor of 10^{-5}. Here we learn all out-scaling factors (see Section 'Learning of weight-to-conductance conversion factors'). We also tested weight remapping (every 250 batches) as well as not ramping up the noise injection, however, results did not improve. Results are averaged over 3 training trials with same hyper-parameters. Finally, we randomly select 1% of devices to be stuck at $\hat{g}_{\min} = 0$ throughout training to provide some added robustness (i.e. drop-connect [7]).

A.1.2 Speech-to-text LSTM with HMM

An automatic speech recognition DNN based on an hidden Markov model (HMM) acoustic model on the Switchboard (SWB) 300 dataset [8] is used. The training set consists of 262 hours of SWB 1 audio with transcripts provided by the Mississippi State University. The test set is the Hub5 2000 evaluation set composed of two parts: 2.1-hour SWB data from 40 speakers and 1.6-hour call-home (CH) data from 40 speakers. The acoustic model is a 4-layer bidirectional LSTM network with input size of 140 and 512 hidden units per direction. On top of the LSTM layers, there is a linear projection layer with 256 hidden units followed by a Softmax output layer with 32K units corresponding to context-dependent HMM states. The acoustic model has a total of 30M trainable weights.

We train the acoustic model in a HWA manner for 30 epochs while monitoring the validation loss, using an 8-bit FP trained model [9] as a starting point. We use an SGD optimizer with momentum of 0.9, batch size of 256 (128 on each of the two GPUs), gradient clipping of 10, dropout of 10%, initial learning rate of 0.005 which drops by $\sqrt{10}$ at epochs 12 and 24, and inject the expected PCM noise (at $t = 20$s without drift) scaled by a factor of 2 each (sub) mini-batch of a single GPU (2.5 or 3.0 did not improve the results). Noise injection strength was ramped up over the first 10000 batches and weights were remapped every 2500 batches. All out-scaling factors are learned. We also initialized the input-scale to 9.0 to avoid overly small inputs for any of the tiles. The best hyper-parameters for the HWA trained network are based on the average of 3 training trials. In this DNN it turned out that knowledge distillation using the FP model as a teacher for the output layer significantly improves the accuracy of the HWA network. The testing is performed by pushing the input features from test-utterances through the HWA acoustic model, simulated as being implemented in AIMC, to get their posteriors. Thereafter, the HMM-based decoding network – assumed to be implemented in a conventional digital processor without any analog nonidealities – is run on the posteriors to compute the word error rate.

A.1.3 BERT base: GLUE

We evaluate the BERT base model [10] on the General Language Understanding Evaluation (GLUE) benchmark [11], which nominally comprises 9 tasks. However, we excluded one task (WNLI) due to the unusual construction of the data set and the small size of the test set. Therefore, we evaluate 8 GLUE tasks: RTE, STS-B, MRPC, CoLA, SST-2, QNLI, QQP, and MNLI. All linear layers of the 12 layer transformer are assumed to be deployed on AIMC. In a previous study, we examined the effect of integer precision on the attention computation [12]. However, here we assume that the activation multiplications in the attention is done in FP accuracy in digital. We do not train the BERT model from scratch but instead only fine-tune the pre-trained BERT model using HWA training techniques. We use a maximum sequence length of 128, because the length of the vast majority of samples are within 128. During fine-tuning, we scan a variety of hyper-parameters, e.g., learning rate from $5 \cdot 10^{-5}$ to 10^{-3}. We use a batch size of 5 and fixed-value weight clipping. Dropout is not used because it was not found to benefit accuracy. The number of fine-tuning epochs varies from 5 to 20, depending on the size of the data-set for each GLUE task. By using a trained FP model as a starting point, the HWA model can achieve iso-accuracy with the results obtained using standard FP fine-tuning as a reference on most GLUE tasks. We used our retrained FP accuracy scores as the baseline as they are slightly

improved from the values in the original study (average over the 8 GLUE tasks was 17.47%). Our accuracy results are reported on the validation data set.

For the sensitivity analysis (Manuscript Fig. 4, and Manuscript Fig. 5, and the additional analysis in the supplement) we selected one of the 8 GLUE tasks (MRPC) to reduce the number of simulations. In this task, we trained and averaged 3 times, used batch size 20, PCM noise injection ($t = 20$s without drift) with scale 3.0 (4.0 did not improve results), ramping up the injection strength over the first 1400 batches and trained for 40 epochs. Initial learning rate was 2×10^{-3} with a 10× reduction at epoch 23. We did not use IR-drop nor weight decay during HWA training, but learned all output scales and used 0.1% of drop-connect probability.

A.1.4 Albert base: GLUE

The Albert base model is structurally identical to the BERT base model, however, it shares the weights across the 12 layers and thus has a much smaller number of total parameters. Similar to the BERT model, we evaluate the Albert model on the same 8 GLUE tasks, by fine-tuning the pre-trained Albert model. Similarly hyper-parameters are scanned to identify the best parameters for the highest accuracy, e.g., learning rate in the range from $5{\cdot}10^{-5}$ to $1.5{\cdot}10^{-3}$. Batch size is 10 and no dropout is used. We used our retrained FP accuracy scores as the baseline (average over the 8 GLUE tasks was 19.46%).

As for the BERT model, the sensitivity analysis was done on one selected GLUE task (MRPC) to reduce the number of simulations. In this tasks, we used the same hyper-parameter setting as described for BERT, except that batch size was 25 and base learning rate $2.5{\cdot}10^{-3}$. The best injected weight noise scale was 2.0 (we also tested 2.5 or 3.0). Results are averaged over 3 training trials.

A.1.5 ResNet-32: CIFAR10

We used the very same ResNet-32 DNN with slightly non-standard channel setting as defined in the paper [5] and the CIFAR10 DataSet [13]. Additional FP training was done starting from the trained model obtained via ONNX export from the authors of [5], reaching slightly improved accuracy (50 batch size; 2 steps learning rate (LR) schedule with final LR of 1% of the starting LR 0.025; first 2 epochs having 20 times reduced LR warmup; 600 epochs training with cutout augmentation; random cropping and mirroring). Using the same LR scheduling (but different base LR), our standard HWA training was performed starting from the same ONNX model, where we assumed that all layers are computing in AIMC. Weights were remapped twice per epoch (every 500 batches) and injected PCM weight noise ($t = 20$s without drift) ramped up over the initial epochs (20000 batches). The best final injected weight noise scales was selected (from values of 3, 4, and 5). Training was repeated for 3 trials and average results are reported. LR was optimized among for 3 values (5, 2.5, 7.5 $\cdot 10^{-3}$) after confirming in broad test runs that this a good range. Weight and bias decay was set to 0.001 and auto weight scaling and all out-scaling factors are learned (see 'Learning of weight-to-conductance conversion factors').

A.1.6 WideResNet-16: CIFAR100

We trained a WideResnet-16 [14] on the CIFAR-100 dataset [13] using standard floating point training (using random resize, flipping, and cutout augmentation). Then we used our standard HWA-training as described in 'AIMC hardware aware DNN training' assuming all layers in AIMC. HWA training was done for 80 epochs, with a brief warmup (500 batches with 20× reduced LR) and two LR reductions by a factor of 10 (at epochs 38 and 76). Weights were remapped roughly once per epoch (2000 batches; batch size 25), injected PCM weight noise ($t = 20s$ without drift) is ramped up over the initial epochs (40000 batches), and the best of final weight noise scale of 3 and 4 was selected (based on the average of 3 training trials). We further used auto-weight scaling, and learned all out-scaling factors. Additionally, we used bias and weight decay (10^{-3}), and 1% drop connect [7], and selected the best of 4 learning rates (0.05, 0.02, 0.01, and 0.005). Finally, we used distilling of the last activation

layer with the floating point model during HWA training which significantly improved the results in this DNN.

A.1.7 ImageNet DNNs

We used 4 standard architectures for the ImageNet data set [15]: Resnet18 and Resnet50 [16], WideResnet-50 [14], and Densenet-121 [17]. The main difference between the image classification DNNs are the various layer depths and widths. As a starting point, we used a fully trained DNN (using floating point) that is provided with the "torchvision" package [18]. We then re-trained the DNNs in HWA manner as described in 'AIMC hardware aware DNN training'. First and last layers were assumed to be computed in digital and all others in AIMC. The injected weight noise scale (scaling the expected PCM noise at $t = 20$s without drift) and the LR were treated as a hyper-parameters. We reduced the learning rate by a factor 10 of after the initial 10 epochs. Batch size was adjusted to fit into the GPU memory (around 50, see Supplementary Table A.1.8). Two GPUs were used per training run, where the injected weight noise was different for each sub-batch in individual GPUs. Due to computing time constraints, HWA training was limited 15 epochs[1] and a couple of hyper-parameter settings (e.g. noise strength scales 3, 4 and learning rates $2{\cdot}10^{-5}$, and 10^{-5}; all combinations are three times simulated). The best average setting for the 3 training trials (typically, LR 10^{-5} and noise scale 3.0) was selected based on a good compromise between short PCM drift accuracy and long-term stability. Results in Manuscript Table 3 are presented for the very same hyper-parameter setting for all drift times. Auto-weight scaling was used and all out-scalings were learned. We further ramped up of the weight noise strength of the initial epoch (50000 batches) and occasionally remapped the weights to the out scales (every 10000 batches). We also experimented with distilling, which in some cases performed well, however, the final results were obtained without distilling since results were found to be more consistent. Finally, we used a small weight decay (10^{-5}) and a small weight drop connect probability of 0.1% ([7]), and turned off the IR-drop during training (but not during inference) as its effect on HWA training was minimal.

A.1.8 RNN-T speech-to-text

The architecture consists of an acoustic encoder (transcriptor network), a decoder (prediction network), and a joint network. The encoder comprises 6 bi-directional LSTM layers and a fully connected (FC) layer. Input size to the recurrent neural network (RNN) is 340 (including 100-dimensional i-vectors [19]), hidden size is 640. The prediction network consists of an embedding layer (dictionary size 46, embedding vector size 10), a single unidirectional LSTM layer with 768 cells, and a FC layer. Encoder and prediction network outputs are combined multiplicatively in the joint network, with a tanh activation function, and an additional FC layer and a final log-softmax over 46 characters.

HWA training performs fine-tuning from a model with the same architecture, pre-trained for 20 epochs on the audio and character-level transcripts from the Switchboard-1 Telephone Speech Corpus (262 h of audio, [8]), further augmented with speed and tempo perturbation [20], SpecAugment [21], and Sequence Noise Injection [22]. The pre-trained model achieves 11.8% average Word Error Rate (WER) on the Switchboard and Call-Home test sets of the NIST Hub5-2000 evaluation.

The HWA model is trained for few (1-5) epochs on audio sequences of maximum length capped at 500 frames, using SGD with momentum as optimizer, with learning rate 10^{-3} and the methods described in (see 'AIMC hardware aware DNN training'). The injected weight noise was expected PCM noise ($t = 20$s without drift) with a scale set to 1. Here, neither down-scaling nor auto-scaling was used, and only the column-wise out-scaling factors were trained. IR-drop was turned off during training. Dropout fraction was set to 25% and batch size was set to 8 per GPU (4 GPUs with separate injected weight noise). To save GPU memory in this large model, the forward-pass was recomputed when doing the backward pass. See Supplementary Table A.1.8 for a listing of all other hyper-parameter.

[1] In case of WideResnet-50, we extended the training to 75 epochs for the last LR reduction step, which however did not change the train error significantly beyond epoch 15

DNN	#epochs	base LR	batch	noise	decay	w-drop	mom.	clip
ResNet-32 CF10	600	$7.5{\cdot}10^{-3}$	50	3	10^{-3}	-	0.9	-
WideResNet-16 CF100	80	$2{\cdot}10^{-2}$	25	3	10^{-3}	1	0.9	-
ResNet-18 ImNet	15	$4{\cdot}10^{-5}$	2×25	3	10^{-5}	0.1	0.99	-
ResNet-50 ImNet	15	$4{\cdot}10^{-5}$	2×25	3	10^{-5}	0.1	0.99	-
DenseNet-121 ImNet	15	$4{\cdot}10^{-5}$	2×25	3	10^{-5}	0.1	0.99	-
WideResNet-50 ImNet	15	$4{\cdot}10^{-5}$	2×25	4	10^{-5}	0.1	0.99	-
BERT-base GLUE	5-20	$5{\cdot}10^{-5}$-10^{-3}	5	1-3	-	-	0.9	-
BERT-base MRPC	40	$2{\cdot}10^{-3}$	20	3	-	0.1	0.95	5
Albert-base GLUE	5-20	$5{\cdot}10^{-5}$-$1.5{\cdot}10^{-3}$	10	1-3	-	-	0.9	-
Albert-base MRPC	40	$2.5{\cdot}10^{-3}$	25	2	-	0.1	0.95	5
Speech SWB300	30	$5{\cdot}10^{-3}$	2×128	2	-	-	0.9	10
LSTM PTB	60	10^{-2}	20	5	10^{-5}	1	0.9	10
RNN-T	$1-5$	10^{-3}	4×8	1	-	-	0.9	10

Supplementary Table 3: **Hyper-parameters for HWA training.** Overview of SGD hyper-parameter used for hardware-aware (HWA) training starting from the FP-trained model. Columns: 'base LR': largest learning rate then reduced in 1-3 steps over the period of training. 'batch': Batch size per GPU (e.g. 2×10 indicates parallization on 2 GPUs with each batch size of 10). 'noise': scale of the injected noise. 'decay': weight decay. 'w-drop': Drop-connect levels (in %) in addition to the weight noise injection. 'mom.': SGD momentum.'clip': gradient norm clipping. More details are described in the text.

B Supplementary Notes

B.1 Static input range learning versus dynamic scaling

In general it is difficult, if not impossible, to simultaneously achieve perfect utilization of the input activation range, weight range, and output activation range for every input and across every tile in the network.

In our hardware model, we assume that input ranges are statically clipped (and scaled) by a learnable input range α (see Manuscript Fig. 2). This input range is thus fixed and re-determined during inference, and is therefore not dynamically adjusted based on the actual values of each input vector. If dynamically adjusted, the input range might get optimized (eg. very small inputs might not be buried in the noise floor because of a dynamic scaling of the input range), and this could improve the signal-to-noise ratio of the AIMC matrix-vector multiplication (MVM). For instance, it was suggested to scale the input range dynamically by the absolute maximum input value for each input vector to maximize the output range (e.g. so-called noise management in [4]). One drawback of dynamic scaling, however, is that determining the absolute max value of each input vector requires more on-chip digital computation than simply fixing a predetermined scaling factor. Another caveat of enlarging the input range too much is that larger inputs might saturate the output range. In our hardware model, we assume a relatively shallow input-to-output dynamic ratio (IO-ratio), that is we assume 10 fully-on inputs together with 10 fully-on weights (ie. at $g_{\max}$) would saturate the output bound so that larger output values are clipped (compare to Manuscript Fig. 3).

To test whether the trained input ranges in our static approach are sufficiently learned, we turn on the described noise management additionally so that too small inputs would have an improved signal-to-noise ratio (SNR). To avoid a potential bound saturation when testing the effect of the dynamic input scaling, we also turned on the so-called bound management (which uses an dynamic down-scaling of the input only if the output was clipped, see [4]). We found that across DNNs, the improvement in performance (as measured with $\mathcal{A}^{1s}$) is less than 0.1% for all DNNs except for the transformers, where the improvement is more pronounced but still only on the order of 1%. Altogether, we thus conclude that our HWA training method learned the static input range well and could adapt the weight and input code to the shallow IO-ratio assumption across DNNs. A dynamic input scaling mechanism seems not necessary to reach good accuracy for inference when using HWA training approach. However, such an approach might be beneficial to increase the flexibility of models that must be deployed with less or no

HWA training and should also better accommodate reduced bit precision of the input digital-to-analog converter (DAC).

B.2 Generalizing to other memory technologies

Our standard AIMC model is mostly based on general considerations but also includes some aspects that are particular to PCM device technology. In particular, we assumed in Section 'Analog IMC standard MVM model' (1) a conductance dependence short-term weights noise that is tuned to PCM devices, (2) weight programming noise and drift over time that is tuned to PCM devices during inference. When other memory technologies are considered such as resistive random-access memory (ReRAM), these two aspects should be replaced by e.g. ReRAM measurement data to model the inference nonidealities correctly.

Note that other nonidealities such as the quantization, weight and input range limitations, as well as the system noise are general and will not depend on the device technology but are more general characteristics of the hardware design choices.

In the following, we use the published ReRAM measurements of [23] to adapt our standard model to ReRAM. Instead of the PCM drift, ReRAM shows a relaxation process after weight programming that is measured as a conductance change dependent on the initially programmed conductance state (see Extended Data Figure 3d in [23]). We fitted the provided data (at 1 day) with a 4-th order polynomial and replaced the programming error coefficients (see Manuscript Eq. 7) with the fitted values and set $g_{\max} = 40\ \mu\text{S}$, which resulted in a good fit with $c_0 = 0.701\,\mu\text{S}$, $c_1 = 22.086\,\mu\text{S}$, $c_2 = -50.7\,\mu\text{S}$, $c_3 = 47.09\,\mu\text{S}$, and $c_4 = -16.458\,\mu\text{S}$. Similarly, the 1 second ReRAM data could be fit with the polynomial coefficients $c_0 = 0.348\,\mu\text{S}$, $c_1 = 16.030\,\mu\text{S}$, $c_2 = -43.853\,\mu\text{S}$, $c_3 = 45.393\,\mu\text{S}$, and $c_4 = -16.815\,\mu\text{S}$ (see Supplementary Figure 1 A).

Since the ReRAM measurement is an effective weight error that combines programming noise, conductance relaxation, and 1/f noise, we here omit[2] the explicit equations for the other noise sources in case of PCM, such as drift (see Manuscript Eq. 8 and Manuscript Eq. 10). Since no mean drift is expected, we also do not use any drift compensation for ReRAM.

While less data is available for short-term ReRAM read noise, we found from [24] that a conductance-independent short-term read noise of 1% of $g_{\max}$ seems to fit the data well. Finally, we assume the most chip-area efficient design of one device pair per weight (representing positive and negative part of the weight, respectively) as we did in case of the PCM standard AIMC model.

B.2.1 ReRAM-inference model results

We first used the very same HWA trained DNNs and applied the above ReRAM-based AIMC model during the evaluation phase. Results are shown in Supplementary Table 4. We find that also for the ReRAM model, accuracy drop is less than 10% for all models, while the most robust topologies (e.g. RNNs) are still at iso-accuracy (less than 1% normalized accuracy drop in relation to the FP baseline, here measured at 1 day). We like to emphasis again that we here focus on the quality of the HWA pre-training methods, and thus do not consider any (possibly costly) in-place fine-tuning methods which will further improve the accuracy as suggested by [23].

Interestingly, we find that robust models to PCM are generally also robust when evaluated on ReRAM (Spearman rank-correlation of normalized accuracy at 1 day is 81%) suggesting that noise robustness largely does not depend on the device technology but on the DNN topology.

In particular, as for PCM (see Manuscript Table 3), accuracy drop is largest for larger convolutional neural networks (CNNs) (using ImageNet). Note that that the ReRAM accuracy is generally somewhat lower at comparable times (1 day) as can be expected by the slightly higher noise measured in particular for smaller conductance values (see Supplementary Figure 1 B). However, here only one type of ReRAM and PCM device is measured and it can therefore not concluded that one technology is better than the other, but rather that larger weight noise, in particular in a conductance region that is overlapping

[2]In the AIHWKit, we can simply use set the programming `coeffs` and `g_max` accordingly and turn off the read noise and drift.

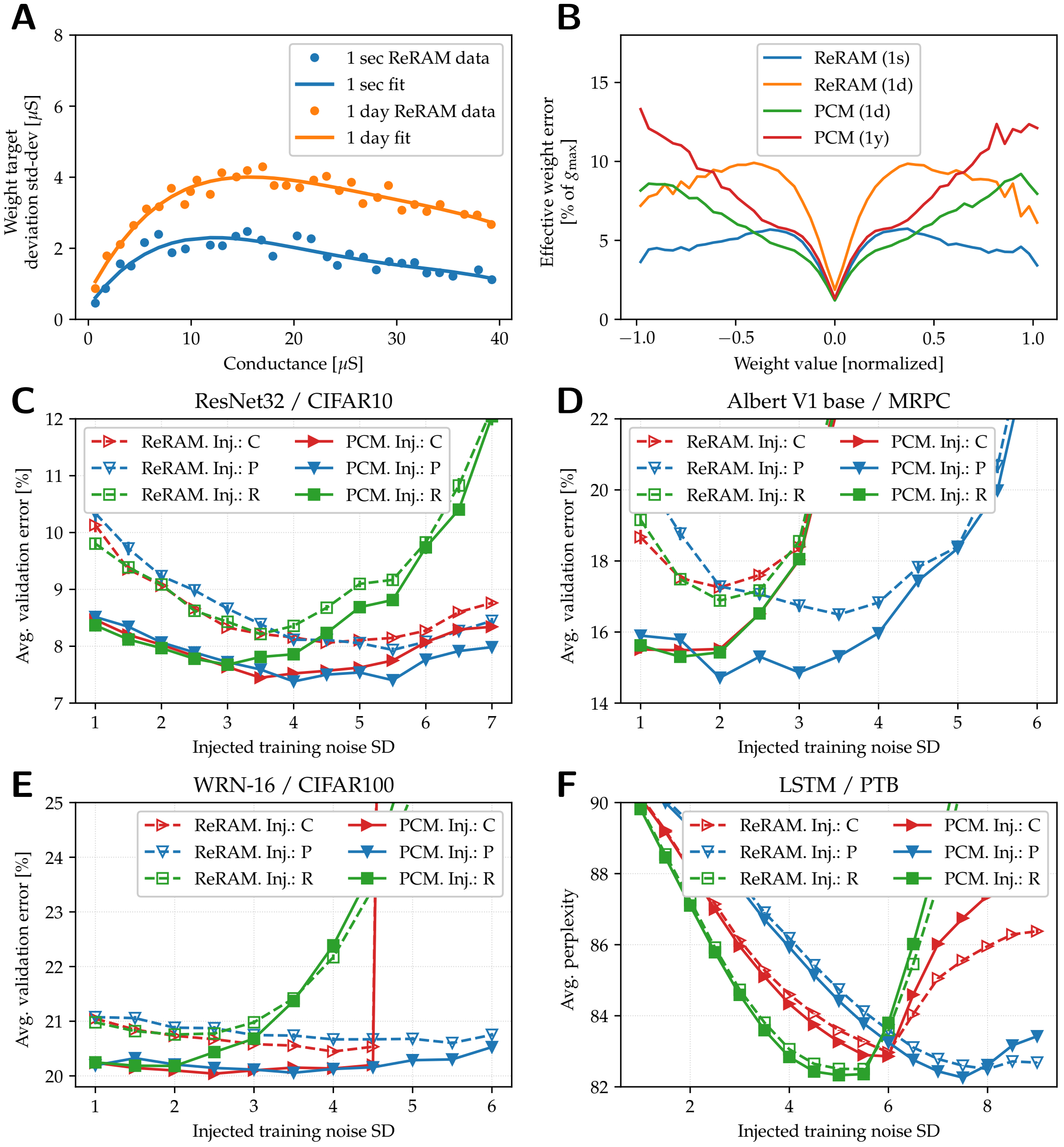

Supplementary Figure 1: **Generalization of the AIMC model to ReRAM.** **A** Measured programming noise and temporal relaxation data for ReRAM synapse extracted from [23] (cf. Extended Data Figure 3d). Data were fit to a 4-th order polynomial so that Manuscript Eq. 7 can be extended to model ReRAM. **B** Effective weight error for a random matrix using the full analog in-memory computing (AIMC) model with PCM or ReRAM weight noise models. The AIMC weight is extracted by estimating a linear operator from random inputs and AIMC outputs and then binning for the weight value. **B**, **C** Study on whether the type of injected weight noise distribution improves for the different weight noise structures in case of PCM and ReRAM. Here ResNet-32/CIFAR10 (**C**) or Albert / MRPC (**D**) is trained for many times with different noise strengths and statistical structure (injected noise types: 'C': constant, 'P': PCM programming noise coefficients, 'R': ReRAM programming noise coefficients at 1 sec, see A). This noise is injected during HWA training. The trained models are then evaluated using either PCM-based (filled symbols) or ReRAM-based (open symbols) AIMC-MVM model. Interestingly, injection of PCM-like noise seems to result in more robust models for inference on both PCM or ReRAM, suggesting that the injection noise structure is beneficial for model convergence independent of the noise structure during evaluation. Results are averaged over three training trials per DNN and injected noise setting, and over 25 inference trials and standard error of the mean is indicated with vertical bars.

**ReRAM HWA-t**	Test Error in %			Norm. Acc. in %
DNN	FP_{32}	1 sec	1 day	$\mathcal{A}_*^{1d}$
ResNet-32 CF10	5.80	6.73 0.02	8.09 0.08	97.3
WideResNet-16 CF100	20.00	19.76 0.02	20.62 0.03	**99.2**
ResNet-18† ImNet	30.50	31.74 0.03	34.92 0.08	93.6
ResNet-50† ImNet	23.87	25.00 0.02	27.64 0.06	95.0
DenseNet-121† ImNet	25.57	26.97 0.03	31.23 0.08	92.4
WideResNet-50† ImNet	21.53	23.74 0.02	25.29 0.03	95.2
BERT-base MRPC	14.60	13.94 0.11	15.10 0.11	**99.0**
Albert-base MRPC	15.08	15.30 0.11	17.54 0.15	95.2
Speech□ SWB300	14.05	14.26 0.02	14.47 0.02	**99.5**
LSTM PTB	72.90	72.97 0.00	73.05 0.00	**99.5**
RNN-T SWB300	11.80	12.37 0.04	13.48 0.08	98.1

Supplementary Table 4: **Inference of using ReRAM-based AIMC crossbar model.** Test error in % ± standard error of mean (across 10-25 inference repeats per training trial and up to 3 training trails) for DNN deployment on analog in-memory computing (AIMC) crossbars after HWA training evaluated using ReRAM data for 1 second and 1 day after programming. Rightmost column shows the normalized accuracy, scaled between the FP reference and chance error, for 1 day after weight programming. Note that ReRAM relaxation here is from[23] data which only provides measured weight errors for up to days after programming. HWA trained DNNs are identical to that used for Manuscript Table 3, only the evaluation model is adapted to ReRAM. BERT and Albert results are here only evaluated on the MRPC GLUE task; SWB300 results are averaged over two benchmark tasks.

with the weight distribution, will result in a higher accuracy drop. Large CNNs seem in particular sensitive (which is in agreement with the sensitivity analysis of Manuscript Fig. 4).

B.2.2 HWA-training with different injected weight noise

In our HWA-training, we found that injecting weight noise with (scaled) statistical structure of the expected PCM noise resulted in robust model on our PCM-based AIMC standard model. Since we thus used the weight noise structure expected during PCM-based inference, the HWA-trained DNNs of Manuscript Table 3 might be sub-optimal for an ReRAM-based inference. To evaluate the effect of the injected weight noise structure, we re-trained a selection of the DNNs (ResNet-32/CIFAR10, WideResNet-16/CIFAR100, AlBERT/MRPC, LSTM/PTB) with various different distributions of injected weight noise, namely PCM-programming noise[3], ReRAM programming noise, or uniform noise (Supplementary Figure 1, summarized in Supplementary Table 5). We found that PCM programming noise seems actually to be superior to either ReRAM or constant noise, even when evaluated on ReRAM-based noise (see Supplementary Table 5, bold values). This might be caused by an improved HWA training convergence with the more multiplicative structure of the PCM noise (ie. increased noise for larger weights). However, simply constant noise is very close to the PCM programming noise, too, so that it could serve a first choice for new memory technologies as well. This is in line with the results of [5], which also showed that the statistical structure of injected weight noise is not too important. However, since we find that PCM is equally good or even slightly superior to the other noise injection in the DNNs tested, we conclude that the ReRAM results are likely not negatively affected by the PCM-like weight noise assumptions during the HWA-training in Supplementary Table 4.

**Noise injection**		**ReRAM** (1 day) Test Error in %			**PCM** (1 day) Test Error in %		
DNN	FP_{32}	Inj.: C	Inj.: P	Inj.: R	Inj.: C	Inj.: P	Inj.: R
ResNet-32 CF10	5.80	8.06 $_{0.04}$	**7.93** $_{0.04}$	8.21 $_{0.05}$	7.45 $_{0.03}$	**7.38** $_{0.03}$	7.68 $_{0.05}$
WideResNet-16 CF100	20.00	**20.45** $_{0.03}$	20.60 $_{0.03}$	20.76 $_{0.03}$	**20.04** $_{0.02}$	20.06 $_{0.02}$	20.18 $_{0.03}$
Albert-base MRPC	15.08	17.25 $_{0.14}$	**16.49** $_{0.14}$	16.89 $_{0.12}$	15.49 $_{0.14}$	**14.72** $_{0.11}$	15.30 $_{0.14}$
LSTM PTB	72.90	72.99 $_{0.00}$	**72.95** $_{0.00}$	72.97 $_{0.01}$	72.96 $_{0.00}$	**72.93** $_{0.00}$	72.95 $_{0.01}$
Mean [norm. acc.]	100.0	98.04	**98.43**	98.09	99.24	**99.67**	99.23

Supplementary Table 5: **Weight injection noise distributions.** Effect of different noise injection types during HWA training for a selection of DNNs. Weight noise injection type is either constant noise (C), PCM programming noise statistics (P) or ReRAM programming noise statistics (R). Here, same data is summarized but the best noise strength is selected from Supplementary Figure 1 C-F. Inference is done either with the ReRAM or PCM-based AIMC model at 1 day. Interestingly, except for the WideResNet-16 model, PCM-like noise injection leads to the smallest test error (in bold) on both AIMC inference choices, suggesting the particular noise structure is beneficial independent of the device technology (last row shows the average normalized accuracy $\mathcal{A}_*^{1d}$ across the DNNs)

**Direct mapping** γ	Test Error in %					Norm. Acc. in %		
DNN	FP_{32}	1 sec	1 hour	1 day	1 year	$\mathcal{A}_*^{1h}$	$\mathcal{A}_*^{1d}$	$\mathcal{A}_*^{1y}$
ResNet-32 CF10	5.80	13.35 $_{0.33}$	15.89 $_{0.48}$	17.54 $_{0.60}$	30.00 $_{0.96}$	88.0	86.1	71.3
WideResNet-16 CF100	20.00	25.73 $_{0.18}$	28.02 $_{0.27}$	32.32 $_{0.41}$	63.93 $_{0.75}$	89.8	84.4	44.4
ResNet-18† ImNet	30.50	47.35 $_{0.40}$	57.15 $_{0.64}$	69.78 $_{0.83}$	95.06 $_{0.25}$	61.6	43.4	7.0
ResNet-50† ImNet	23.87	51.87 $_{0.64}$	82.09 $_{0.64}$	98.24 $_{0.08}$	99.85 $_{0.00}$	23.4	2.2	0.1
DenseNet-121† ImNet	25.57	54.83 $_{0.76}$	76.63 $_{0.81}$	95.81 $_{0.27}$	99.80 $_{0.01}$	31.3	5.5	0.1
WideResNet-50† ImNet	21.53	48.36 $_{0.33}$	92.89 $_{0.25}$	99.66 $_{0.01}$	99.89 $_{0.00}$	8.9	0.3	0.0
BERT-base GLUE8	17.47	33.45 $_{0.19}$	33.77 $_{0.20}$	33.32 $_{0.24}$	32.85 $_{0.20}$	65.3	66.3	67.3
Albert-base GLUE8	19.46	32.00 $_{0.00}$	32.00 $_{0.00}$	31.25 $_{0.00}$	31.50 $_{0.00}$	72.2	73.8	73.3
Speech□ SWB300	14.05	15.36 $_{0.03}$	17.63 $_{0.03}$	22.03 $_{0.05}$	53.26 $_{0.31}$	95.8	90.7	54.4
LSTM PTB	72.90	73.94 $_{0.02}$	74.82 $_{0.02}$	76.49 $_{0.02}$	82.11 $_{0.03}$	92.9	86.8	66.0
RNN-T SWB300	11.80	13.83 $_{0.03}$	17.67 $_{0.21}$	43.74 $_{1.49}$	91.65 $_{0.18}$	93.3	63.8	9.4

Supplementary Table 6: **Inference results for FP-trained DNNs using tile-wise output scales.** Same direct mapping of the FP-trained DNNs as described in Manuscript Table 2, except that a tile-wise output scalar γ is assumed (instead of the output-wise γ_i). The weights are mapped to conductances by setting the absolute max value to $g_{\max}$ and thus $\gamma = \max_{ij} |w_{ij}|$. Note that in comparison with Manuscript Table 2, the results are much inferior, indicating the benefit of having output-wise digital scales to further improve the compactness of conductance values distribution (see also Manuscript Fig. 6).

B.3 Tile-wise digital instead of column-wise digital output scale

In our AIMC standard model, we assume that each crossbar array has configurable digital (FP) output scales, one per output column, which we learn in our approach during HWA training. Whether to have these FP scales available is a chip design choice. However, for the broad range of DNN topologies investigated here, it is necessary to have at least some capability of scaling the ADC output ticks back to the original floating point range, since the networks have a number of non-linear computations that need to be done in FP, such as non-linear activation functions (GeLU for the Transformers, Sigmoid and Tangent for LSTMs) and other digital computations such as attention, layer-norm, or Softmax. Thus, conversions from ADC integer values to FP as well as the capability of on-chip auxiliary FP computations are essential when aiming to support DNNs of various topologies and thus need to be

[3]In this experiment, we only used the PCM programming noise distribution (ie. expected PCM noise at $t = 0$s) and did not include the 1/f read-noise at 20 sec as done for the main results Manuscript Table 3 since we do not have similar data for ReRAM. However, the shape of the distribution does not change significantly by including the 1/f noise. In fact, one can approximately obtain the distribution with read noise by simply scaling the PCM programming noise by about 1.2. Thus, since we widely varied the noise scale in Supplementary Figure 1 D-F, this approach here does not differ significantly from the approach in the main text.

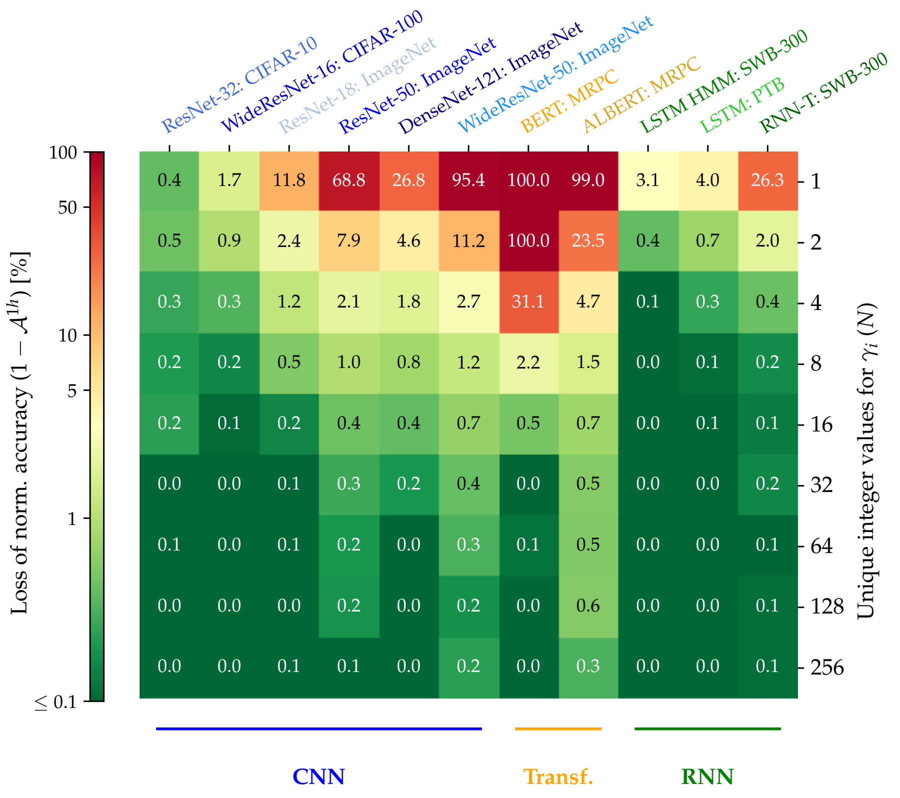

Supplementary Figure 2: **Compression of column-wise output scales**. HWA trained DNNs in Manuscript Table 3 were trained with the chip-design assumption of having a programmable FP scale per AIMC output (ie. per ADC). Here we test the impact, if the scales are represented by N integer numbers and one tile-wise scale (see Manuscript Eq. 1). Loss of accuracy in relation to when γ_i are in FP precision is shown in color code for 1 day PCM drift. Note that simply multiplying the scale values into the conductances (that is $N = 1$) performs poorly in particular for larger DNN. However, 4-bit integer values ($N = 16$) are already enough for all DNNs when one would require a less than 1 % drop in (normalized) accuracy.

available on the chip.

Whether separate γ_i per output column (see Manuscript Eq. 21) or rather a tile-wise scalar value γ is available is a further design choice. While the number of FP multiplications is identical, the amount of on-chip storage capacity needed in the former case is higher. In practice, however, since each ADC (assuming parallel ADCs, one per output column) have slightly different characteristics, a programmable FP scale per column can also be used to calibrate the ADC and minimize the ADC-to-ADC variations (see e.g. [25]). Moreover, column-wise output scales serve another dual-use: many linear or convolution layers of modern DNNs are followed by a batch-norm or layer-norm layer, which typical require (at least) a scaling operation per output column, where these γ_i could be re-used again (by combining all scaling factors) to save additional computations and loading operations.

From the obtainable accuracy point of view, we find that column-wise scales significantly improve the direct mapping of FP-trained DNNs to AIMC, as shown by the vastly inferior results in Supplementary Table 6 (using just a tile-wise scale) in comparison to the approach described in the main text (see Manuscript Table 2). This improved initial mapping also means that the column-wise output scales reduce the effort of HWA training significantly as the retraining starts from an much improved conductance distribution as we show in the section 'Training with column-wise versus tile-wise scales'.

In the next section, we show that incorporating additional digital scales (e.g. batch norm or the learned γ_i in our approach) into the AIMC weights after HWA training in retrospect (e.g. for saving of the additional FP computations or reducing on-chip memory requirements) is typically not a good

**Digital scale(s)**			Test Error in %			
DNN	scales	# hyp.	1 sec	1 hour	1 day	1 year
BERT-base MRPC	γ	72	14.27 0.10	14.62 0.09	14.58 0.12	15.92 0.11
BERT-base MRPC	$\{\gamma_i\}$	4	13.95 0.09	14.15 0.09	14.47 0.11	**15.40** 0.13
Albert-base MRPC	γ	234	14.61 0.12	15.28 0.12	15.40 0.15	16.76 0.16
Albert-base MRPC	$\{\gamma_i\}$	6	15.13 0.10	15.42 0.12	15.57 0.11	**16.33** 0.10

Supplementary Table 7: **Training with tile-wise output scales.** Repeat of the HWA training for the transformers on MRPC with either allowing for digital FP output scales per output (γ_i) or only a single scale for each crossbar tile (γ). In both cases, SGD is used to optimize the scale(s) during HWA (as apposed to Supplementary Figure 2, where scales are changed post HWA training). Training with only a single scale is possible and results are comparable overall, however, the number of hyper-parameters tested here was much higher to achieve adequate results (# hyp), indicating that HWA training is much more challenging with only a single scale per tile. Here, the hyper-parameters that resulted in the best average accuracy across drift times is taken and reported, averaged over 3 training trials. Even with much less hyper-parameter combinations tested, the long-term (1 year) accuracy is actually better for the per-output scale case (indicated in bold), showing its superior robustness.

idea in particular for large DNNs. However, as we show also in the following analysis, the (512) values $\gamma_i > 0$ can be typically represented by only 4-bit integer numbers (plus a single FP tile-wise scale) without significantly impacting the accuracy and without re-training, which would significantly reduce the additional memory requirements[4].

B.3.1 Integer precision for output scales to save memory

Assume we find by HWA training a (normalized) conductance matrix weight $\breve{W}$, which represents the original weight W together with the column-wise output scales, ie. $W \approx \text{diag}\gamma_i \breve{W}$. We can discretize the output scales to N integer values times a scalar FP value by setting $W \approx \text{diag}q_i \left(\text{diag}\frac{\gamma_i}{q_i} \breve{W}\right)$, that is, modifying the conductance values to correct for the difference of γ_i and the approximations q_i. Here we ensure that $q_i \geq \gamma_i$ to not increase the conductance values beyond $g_{\max}$. We set $\alpha_N = \frac{\max_k \gamma_k}{N}$ and define

$$q_i = \alpha_N \left\lceil \frac{\gamma_i}{\alpha_N} \right\rceil \tag{1}$$

with at most N integer values $\left\lceil \frac{\gamma_i}{\alpha_N} \right\rceil$ ranging from $1, \ldots, N$.

In Supplementary Figure 2 we show the impact of different settings of the number of unique scale values N on the inference accuracy (at 1 day PCM drift) of the HWA trained DNNs of Manuscript Table 3. In the extreme case of $N = 1$, i.e. just having a single scale α_1 per tile, the accuracy is impacted severely in some DNNs, in particular for the transformers. This also indicates that accuracy will be highly impacted if other scales such as the batch-norm scales would be multiplied into the conductance values without retraining. However, if one allows for as little as $N = 16$ integer values (4-bit) representing the up to 512 γ_i values, the impact on the accuracy is less than 1% across all DNNs, thus reducing greatly the required memory for the γ_i in practice.

B.3.2 Training with column-wise versus tile-wise scales

While we laid out in the above that having programmable FP scales per output column γ_i is beneficial in multiple ways, and multiplying such scales into the conductance matrix does not work well, one could also try to do the HWA training with only assuming a single tile-wise scale γ to begin with. We tested this approach for the transformers, which fared the worst when setting $N = 1$ in retrospect without retraining (see Supplementary Figure 2).

[4]However, still the ADC calibration scales as well as batch norm factors need to be considered.

DNN	$n_{\mathrm{col}} = 256$	$n_{\mathrm{col}} = 512$	$n_{\mathrm{col}} = 1024$	$n_{\mathrm{col}} = \max$	Smallest $n_{\mathrm{col}} = 1024$
ResNet-18: ImageNet	98.71 0.04	98.92 0.04	**99.03** 0.03	**99.12** 0.01	98.50 0.05
ResNet-50: ImageNet	98.90 0.03	98.86 0.03	98.98 0.02	**99.01** 0.03	98.74 0.04
DenseNet-121: ImageNet	98.52 0.06	98.60 0.05	98.89 0.05	**99.03** 0.01	98.43 0.03

Supplementary Table 8: **PCM noise-exempt columns for ImageNet CNNs.** Similar analysis as in Manuscript Fig. 7, however, here only a subset of columns in the 6 most sensitive layers is made PCM-noise-exempt. Normalized accuracy $\mathcal{A}_*^{1h}$ is shown in percent. The columns are selected based on the average absolute gradients. Note that 1024 columns is enough (or very close) to iso-accuracy, which again about halves the parameter needed to be set to PCM-noise-exempt in the Manuscript Fig. 7 analysis showing that area can still be improved. The last column shows that normalized accuracy if the columns with the least gradient magnitude values are selected instead showing the correlation of this measure with the overall accuracy improvement.

As shown in Supplementary Table 7, we found that while training using a tile-wise scale greatly improved the results in comparison to without retraining (ie. $N = 1$ in Supplementary Figure 2), the best long-term accuracy drop (1 year) is still somewhat higher when compared to training with column-wise scales, indicating its inferior robustness.

However, more importantly in practice, HWA training with only a single tile-wise scale instead of the column-wise scales was more difficult in a sense that we had to scan more carefully hyper-parameters (learning rates, injected noise strengths, weight decay) as well as using Gaussian clipping (instead of simple fixed-value clipping) which has an additional hyper-parameter to tune (its width, see also [5]) and thus further increases the effort.

In summary, since output scales per column can be used also for additional operations such as batch-norms, as well as for ADC calibration, and moreover HWA training effort is significantly reduced while still achieving superior results (since the DNN retraining start with a much more compact conductance distribution, compare to Supplementary Table 6), we thus only consider training column-wise output scales in our HWA training approach in the main text.

B.4 Column-wise noise impact for ImageNet CNNs

In Manuscript Fig. 7, it is shown that turning off the PCM noise of very few layers (equal or less than the 6 most sensitive) are enough to achieve iso-accuracy ($\mathcal{A}_*^{1h} \geq 99\%$). While improving the noise for a full layer is feasible (e.g. by cloning the layer weights onto multiple tiles and computing in parallel and averaging the results), it comes with area cost. Instead of reducing the PCM noise in a full layer one could also attempt to reduce it only for a subset of the columns that are mostly responsible for the sensitivity.

To see whether this would decrease further the area cost, we first estimated the sensitivity of each conductance column of the crossbars in the most sensitive layers of each DNN that could achieve iso-accuracy (ResNet-18, ResNet-50, and DenseNet-121). Altogether the DNNs had 2048, 2048, and 1664 columns, respectively, in the sensitive layers that needed to be set to PCM-noise-exempt to reach iso-accuracy in Manuscript Fig. 7C.

To estimate the sensitivity of each column within these layers, we computed the mean absolute gradient for each of the columns for 100 input batches. We sorted the columns according to these values and picked a subset of columns with the largest values and tested the DNNs accuracy, when only setting those columns to PCM-noise-exempt instead of all columns in the most sensitive layers.

The results of this analysis are shown in Supplementary Table 8. We find that the column selection method indeed correlates with accuracy as selecting the columns with the least average absolute gradient values, results in a much larger accuracy drop than selecting the columns with the maximal values (see Column '$n_{\mathrm{col}} = 1024$' versus Column 'Smallest $n_{\mathrm{col}} = 1024$').

We also find that a subset of 1024 columns (instead of all the columns in the sensitive layers, ie. Column '$n_{\mathrm{col}} = \max$' in Supplementary Table 8) is enough or very close to iso-accuracy thus reducing the area requirements by about half. For 512 or less PCM-noise-exempt columns, accuracy drops more significantly below the iso-accuracy target.

References

[1] M. J. Rasch, D. Moreda, T. Gokmen, M. Le Gallo, F. Carta, C. Goldberg, K. E. Maghraoui, A. Sebastian, and V. Narayanan, "A flexible and fast pytorch toolkit for simulating training and inference on analog crossbar arrays," *IEEE International Conference on Artificial Intelligence Circuits and Systems (AICAS)*, pp. 1–4, 2021.

[2] M. J. Rasch et al., "IBM Analog Hardware Acceleration Kit 0.8.0" , *IBM/aihwkit*, `https://doi.org/10.5281/zenodo.8148598`, 2023.

[3] S. R. Nandakumar, I. Boybat, V. Joshi, C. Piveteau, M. Le Gallo, B. Rajendran, A. Sebastian, and E. Eleftheriou, "Phase-change memory models for deep learning training and inference," *IEEE International Conference on Electronics, Circuits and Systems*, pp. 727–730, 2019.

[4] T. Gokmen, M. Onen, and W. Haensch, "Training deep convolutional neural networks with resistive cross-point devices," *Frontiers in neuroscience*, vol. 11, p. 538, 2017.

[5] V. Joshi, M. Le Gallo, S. Haefeli, I. Boybat, S. R. Nandakumar, C. Piveteau, M. Dazzi, B. Rajendran, A. Sebastian, and E. Eleftheriou, "Accurate deep neural network inference using computational phase-change memory," *Nature Communications*, vol. 11, no. 2473, pp. 1–13, 2020.

[6] A. Taylor, M. Marcus, and B. Santorini, "The Penn Treebank: An overview," 2003.

[7] L. Wan, M. Zeiler, S. Zhang, Y. Le Cun, and R. Fergus, "Regularization of neural networks using dropconnect," in *Proceedings of the 30th International Conference on Machine Learning* (S. Dasgupta and D. McAllester, eds.), vol. 28 of *Proceedings of Machine Learning Research*, pp. 1058–1066, Atlanta, Georgia, USA: PMLR, 17–19 Jun 2013.

[8] J. J. Godfrey and E. Holliman, "Switchboard-1 release 2 ldc97s62," 1993.

[9] X. Sun, J. Choi, C.-Y. Chen, N. Wang, S. Venkataramani, V. V. Srinivasan, X. Cui, W. Zhang, and K. Gopalakrishnan, "Hybrid 8-bit floating point (hfp8) training and inference for deep neural networks," *Advances in neural information processing systems*, vol. 32, 2019.

[10] J. Devlin, M.-W. Chang, K. Lee, and K. Toutanova, "Bert: Pre-training of deep bidirectional transformers for language understanding," *arXiv preprint arXiv:1810.04805*, 2018.

[11] A. Wang, A. Singh, J. Michael, F. Hill, O. Levy, and S. R. Bowman, "Glue: A multi-task benchmark and analysis platform for natural language understanding," *arXiv preprint arXiv:1804.07461*, 2018.

[12] K. Spoon, H. Tsai, A. Chen, M. J. Rasch, S. Ambrogio, C. Mackin, A. Fasoli, A. M. Friz, P. Narayanan, M. Stanisavljevic, and G. W. Burr, "Toward Software-Equivalent Accuracy on Transformer-Based Deep Neural Networks With Analog Memory Devices," *Frontiers in Computational Neuroscience*, vol. 15, no. July, pp. 1–9, 2021.

[13] A. Krizhevsky, G. Hinton, *et al.*, "Learning multiple layers of features from tiny images," 2009.

[14] S. Zagoruyko and N. Komodakis, "Wide residual networks," *arXiv preprint arXiv:1605.07146*, 2016.

[15] J. Deng, W. Dong, R. Socher, L.-J. Li, K. Li, and L. Fei-Fei, "Imagenet: A large-scale hierarchical image database," in *2009 IEEE conference on computer vision and pattern recognition*, pp. 248–255, Ieee, 2009.

[16] K. He, X. Zhang, S. Ren, and J. Sun, "Deep residual learning for image recognition," in *Proceedings of the IEEE conference on computer vision and pattern recognition*, pp. 770–778, 2016.

[17] G. Huang, Z. Liu, L. Van Der Maaten, and K. Q. Weinberger, "Densely connected convolutional networks," in *Proceedings of the IEEE conference on computer vision and pattern recognition*, pp. 4700–4708, 2017.

[18] A. Paszke, S. Gross, F. Massa, A. Lerer, J. Bradbury, G. Chanan, T. Killeen, Z. Lin, N. Gimelshein, L. Antiga, A. Desmaison, A. Köpf, E. Yang, Z. DeVito, M. Raison, A. Tejani, S. Chilamkurthy, B. Steiner, L. Fang, J. Bai, and S. Chintala, "Pytorch: An imperative style, high-performance deep learning library," 2019.

[19] N. Dehak, P. J. Kenny, R. Dehak, P. Dumouchel, and P. Ouellet, "Front-end factor analysis for speaker verification," in *IEEE Transactions on Audio, Speech, and Language Processing*, vol. 19, pp. 788–798, 2011.

[20] T. Ko, V. Peddinti, D. Povey, and S. Khudanpur, "Audio augmentation for speech recognition," in *2015 IEEE International Conference on Acoustics, Speech and Signal Processing (ICASSP)*, pp. 3586–3589, 2015.

[21] D. S. Park, W. Chan, Y. Zhang, C.-c. Chiu, B. Zoph, E. D. Cubuk, and Q. V. Le, "SpecAugment: A simple data augmentation method for automatic speech recognition," in *Proc. Interspeech*, pp. 2613–2617, 2019.

[22] G. Saon, Z. Tüske, K. Audhkhasi, and B. Kingsbury, "Sequence noise injected training for end-to-end speech recognition," in *2019 IEEE International Conference on Acoustics, Speech and Signal Processing (ICASSP)*, pp. 6261–6265, 2019.

[23] W. Wan, R. Kubendran, C. Schaefer, S. B. Eryilmaz, W. Zhang, D. Wu, S. Deiss, P. Raina, H. Qian, B. Gao, *et al.*, "A compute-in-memory chip based on resistive random-access memory," *Nature*, vol. 608, no. 7923, pp. 504–512, 2022.

[24] M. Zhao, H. Wu, B. Gao, Q. Zhang, W. Wu, S. Wang, Y. Xi, D. Wu, N. Deng, S. Yu, H.-Y. Chen, and H. Qian, "Investigation of statistical retention of filamentary analog rram for neuromophic computing," in *2017 IEEE International Electron Devices Meeting (IEDM)*, pp. 39.4.1–39.4.4, 2017.

[25] R. Khaddam-Aljameh, M. Stanisavljevic, J. F. Mas, G. Karunaratne, M. Braendli, F. Liu, A. Singh, S. M. Müller, U. Egger, A. Petropoulos, T. Antonakopoulos, K. Brew, S. Choi, I. Ok, F. L. Lie, N. Saulnier, V. Chan, I. Ahsan, V. Narayanan, S. R. Nandakumar, M. L. Gallo, P. A. Francese, A. Sebastian, and E. Eleftheriou, "HERMES Core – A 14nm CMOS and PCM-based In-Memory Compute Core using an array of 300ps/LSB Linearized CCO-based ADCs and local digital processing," in *Symposium on VLSI Circuits*, 2021.
